# Data on the Validation to Determine the Material Thermal Properties Estimation Via a One-Dimensional Transient Convection Model

**DOI:** 10.1016/j.dib.2021.107632

**Published:** 2021-11-26

**Authors:** Lauren B. Tomanek, Daniel S. Stutts

**Affiliations:** Department of Mechanical and Aerospace Engineering, Missouri University of Science and Technology, Rolla, MO 65409, USA

**Keywords:** Thermal conductivity, Additive manufacturing, Analytical model, Parameter estimation, One-dimensional, Transient analysis

## Abstract

These data were acquired to estimate the parameters of a closed form solution of a one-dimensional transient convection heat diffusion PDE. The purpose was to demonstrate that the model could be used to determine the thermal conductivity. The system was tested for a wide range of thermal conductivity, 15-400 W/mK, in order to verify that the method was applicable for various materials. The data reported here refer to the study in the research articles, “Material Thermal Properties Estimation Via a One-Dimensional Transient Convection Model” [1] and “Influence of porosity on the thermal, electrical, and mechanical performance of selective laser melted stainless steel” [2]. The dataset contains the raw data obtained from the temperature acquisition system as well as the processed results from a Python program to determine the thermal conductivity from a forced convection, transient one-dimensional heat diffusion equation.

## Nomenclature

A: cross-sectional area of the rod
c: specific heat of the rod
d: diameter of the rod
h: heat transfer coefficient
k: thermal conductivity of the rod
$k_{f}$: thermal conductivity of the fluid
L: length of the rod in the domain
Nu: Nusselt number of the fluid
P: steady state power into the rod
Pe: electrical power
Pr: Prandtl number of the fluid
Q: heat flux
Re: Reynolds number of the fluid
s: perimeter of the rod
T: temperature
$T_{\infty }$: ambient temperature
t: time
x: distance along rod from heater
$\left(\dot{\;}\right)$: $\frac{\partial }{\partial t}\left(\right)$
${\left(\right)}^{\prime }$: $\frac{\partial }{\partial x}\left(\right)$

**Greek symbols**
α: heat flow into the boundary
η: input power efficiency
θ: excess temperature
$\theta_{0}^{\prime }$: temperature gradient at the boundary
κ: thermal diffusivity of the rod
ν: diffusion rate coefficient
ρ: density of the rod
τ: heating delay

## Specifications Table

**Table:** 

Subject	Engineering
Specific subject area	Mechanical Engineering/ Heat Transfer
Type of data	Table (Comma Delimited file format)
	Table (Microsoft Excel file format)
	Program (Python file format)
How data were acquired	Temperature data was acquired using custom Arduino board and code.
Data format	Raw
	Analyzed
Parameters for data collection	Conducted using a wind tunnel and experimental rod setup.
Description of data collection	The data was collected using the test set up that is outlined in [1]. The rods were in a wind tunnel to create a uniform laminar flow. The temperature was acquired using a custom Arduino board which also supplied the power to heat the rod.
Data source location	Institution: Missouri University of Science and Technology City/Town/Region: Rolla, Missouri Country: USA
Data accessibility	Raw and analyzed data are given in the following Mendeley repository [3]. Python code is available in the Zenodo database https://zenodo.org/record/5683312[4].
Related research article	[1] L. B. Tomanek, D. S. Stutts, Material thermal properties estimation via a onedimensional transient convection model, Applied Thermal Engineering 184 (feb 2021). doi:10.1016/j.applthermaleng.2020.116362
	[2] L. B. Tomanek, D. S. Stutts, T. Pan, F. Liou, Influence of porosity on the thermal, electrical, and mechanical performance of selective laser melted stainless steel, Additive Manufacturing 39 (2021) 101886. doi:10.1016/j.addma.2021.101886

## Value of the Data

• The dataset is important for validating the mathematical model presented in “Material Thermal Properties Estimation Via a One-Dimensional Transient Convection Model” [1].
• The data are useful for engineers and researchers to establish this method as an alternative to current ASTM standards to identify the thermal conductivity.
• The data can be used to verify other models for heat diffusion in one-dimension.
• The data can be used to develop a method to test materials with unknown thermal conductivity.
• The data can be used as a comparison with other measurements taken at different facilities.
• This dataset and model was applied to evaluate the effect of porosity on the thermal performance in selective laser melted stainless steel additive manufacturing and can be used to evaluate other materials [2], [5].

## 1 Data Description

The raw experimental data is given in the CSV documents is the raw data collected from running the experiment. Each file contains the time and temperature data from each thermocouple, and the power setting for the test using a 10 bit duty cycle PWM controller. The temperature is in the format of excess temperature (θ). There are three sets of data: constant wind speed at different boundary temperatures [1], constant boundary temperature at different wind speeds [1], and additively manufactured stainless steel 304L [2]. The file naming convention for the files at a constant wind speed of 5 m/s is the boundary temperature, material, and test number (i.e. 50ss1 would be for the first test of SAE-304 at 50°C). The file naming convention for the files at a constant boundary temperature of 75°C is material and wind speed (i.e. ss1 would be for the test of SAE-304 at 1 m/s wind speed). The file naming convention for the additively manufactured materials are the build direction (x or y), if it is the more porous variety or not (p or n), and the test number (i.e. xp1 would be the first test for the more porous specimen built along the x direction).

The oneDkhEstimator Python code included with the article is used to analyze the data. This code takes the raw data and performs the parameter estimation routine outlined in Section 2.

Also given are the results from the parameter estimation given in an Excel document produced by the Python code from the stainless steel (SAE-304), aluminum (Al6061-T6), copper (110 Cu-H04), and four additively manufactured rods. The Excel file contains the values calculated using the parameter estimation code in Python and the list of output variables is given in Table 1. The top cell will have the filename of the data analyzed followed by the number of terms used in the Fourier series. The first set of results is the finite difference boundary condition followed by the steady state model results and the results from the transient model. In each model, the estimated parameters are first given followed by the mean standard error for the model then the error for the individual parameters that is calculated according to Section 2.2.5.

**Table 1:** oneDkhEstimator File Output.

Variable
N
**Finite difference boundary model**
temp_grad
alpha
tau
standard_error
temp_grad error
alpha error
tau error
**Steady state model**
temp_grad
h/k_ratio
standard_error
temp_grad error
h/k_ratio error
**Transient model**
h
k
Pss
standard_error
h error
k error
Pss error

## 2 Experimental Design, Materials and Methods

### 2.1 Experimental Setup

**Fig. 1:**
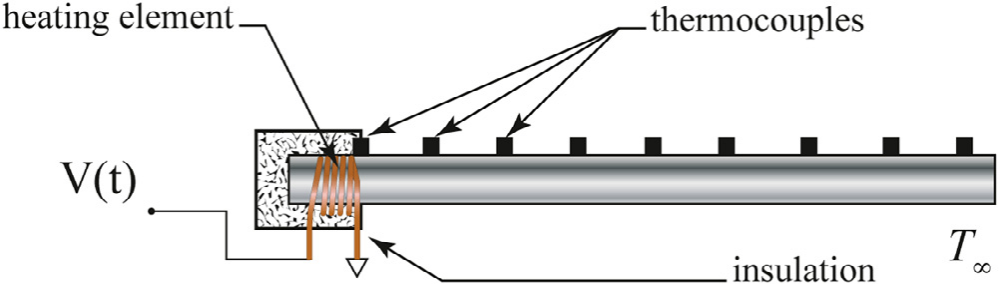
Schematic of Experimental Setup.

Each slender test rod is heated using an open loop control consisting of a resistive heating element made of 34 gauge Nichrome wire. Along the length of the rod are thermocouples used to collect the data used in the parameter estimation. The system is then mounted in the wind tunnel. A diagram of the experimental setup is given in Fig. 1.

### 2.2 Data Processing

The raw data was processed using Python to determine the thermal conductivity. The code will run the parameter estimation routine on the filenames specified, create a plot of the fit of the model to the temperature data, and save the estimated parameters in an Excel document. An overview of the oneDkhEstimator code in Python is given in Listing 1.

**Listing 1:**
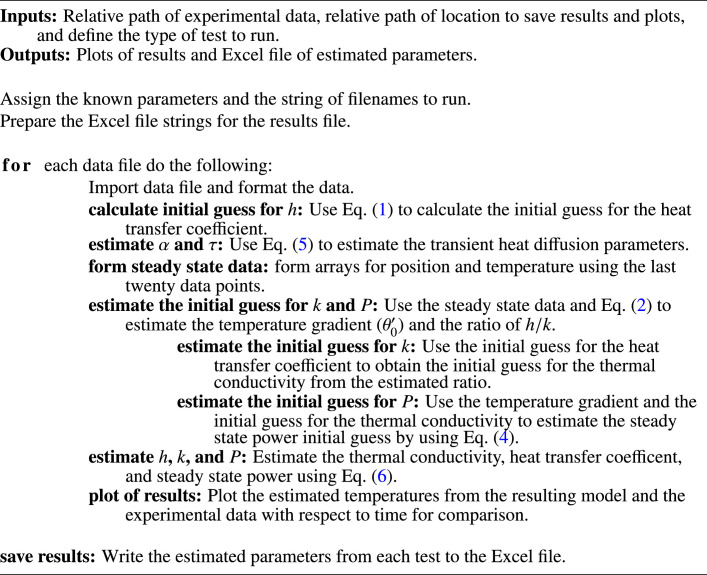
pseudocode of oneDkhEstimator.

The format of the experimental data needed to run the oneDkhEstimator code in Python is a comma delimited text document with columns containing time followed by columns of thermocouple measured temperature data in the order of closest to the heater to farthest away. The program is specified for six thermocouples, but the number can be adjusted in the file preamble. The thermocouple data taken was measured as temperature above ambient also known as the excess temperature.

The code uses the scipy.optimize.least_squares [6] function in Python to estimate the variables using the Levenburg Marquardt algorithm [7]. The convergence criterion used in the Levenburg Marquardt algorithm is detailed on the documentation page [6]. (All of the estimation processes met the convergence criterion, and the quality of fit of the data using Spearman’s rank correlation coefficient is 0.993±0.01 [8].)

#### 2.2.1 Known or Directly-Measured Parameters

The known or directly-measured values for a specific test were: the thermocouple spacing, material density, specific heat, length and diameter of the rod, and wind speed. The number of terms used in the Fourier series in the transient model are specified as well; for this analysis 100 terms are used. Additional parameters given are for formatting of the simulation plots.

#### 2.2.2 Initial Guess Determination for Unknown Parameters

The oneDkhEstimator Python code estimates the thermal conductivity of the materials, but to ensure that the least squares estimation does not diverge from the minimum, the initial guesses are calculated using simpler models. The heat transfer coefficient is the first initial guess that is calculated by using the wind speed and The Churchill-Bernstein equation for flow over a cylinder, Eq. (1) [9].

(1) $$Nu=\frac{hd}{k_{f}}=0.3+\frac{0.62Re^{1/2}Pr^{1/3}}{{\left[1+{\left(0.4/Pr\right)}^{2/3}\right]}^{1/4}}{\left[1+{\left(\frac{Re}{282,000}\right)}^{5/8}\right]}^{-4/5}$$

The steady state solution is able to estimate the ratio of the heat transfer coefficient and thermal conductivity as well as the temperature gradient at the boundary ($\theta_{0}^{\prime }$). The initial guess for the thermal conductivity is estimated by using the ratio in the steady state solution and the initial guess for the heat transfer coefficient found using Eq. (1). The steady state solution is estimated using the last twenty data points from each thermocouple in Eq. (2).

(2) $$\theta \left(x\right)=\frac{\theta_{0}^{\prime }}{m}\left(\frac{\text{cosh}(m(L-x))}{\text{sinh}(\mathrm{mL})}\right)$$

The fin performance factor (m) is given by:

(3) $$m=2\sqrt{\frac{h}{kd}}$$

The initial guess for the power into the rod is calculated by using the estimated value for $\theta_{0}^{\prime }$ and the initial guess for the thermal conductivity in Eq. (4).

(4) $$P=\theta_{0}^{\prime }kA$$

#### 2.2.3 Finite Difference Boundary Condition

The heated boundary condition for the model is used to improve the fit of the model. The boundary defines the flux into the end of the rod. To use the boundary condition, the temperature gradient at the boundary must be known. A second-order forward finite difference approximation is used to approximate the heat flow into the boundary using the temperature data from the first three thermocouples. Using the boundary condition allows for the estimation of the transient heat diffusion parameters, α and τ, using Eq. (5), where Δx is the thermocouple spacing. It was found that first estimating the transient heat diffusion parameters returned a more consistent estimation for the thermal conductivity.

(5) $$\theta_{0}^{\prime }\left(1-e^{-\alpha (t+\tau )}\right)=\frac{3\theta_{1}-4\theta_{2}+\theta_{3}}{2\Delta x}$$

The convergence of the solution is improved by scaling the α and τ parameters in the implementation of this model in order to get all parameters on the same order of magnitude.

#### 2.2.4 Transient Model Analysis

The estimated values for the heat transfer coefficient, thermal conductivity, and steady state power into the rod are used as the initial guesses in the transient model’s parameter estimation. The temperature solution of the model [1] is:

(6) $$\begin{array}{cc} T\left(x,t\right)= & T_{\infty }+\frac{P\left(\nu e^{-\alpha \tau -\nu t}-\nu e^{-\alpha \left(t+\tau \right)}+\left(\nu -\alpha \right)\left(1-e^{-\nu t}\right)\right)}{\mathrm{AcL}\nu \rho \left(\nu -\alpha \right)}\\ & +\;\frac{2P}{\mathrm{AcL}\rho }\sum_{n=1}^{\infty }\left[\left(\frac{e^{-\left(\beta_{n}^{2}+\nu \right)t-\alpha \tau }-e^{-\alpha \left(t+\tau \right)}}{\beta_{n}^{2}-\alpha +\nu }+\frac{1}{\beta_{n}^{2}+\nu }-\frac{\left(\beta_{n}^{2}-\alpha +\nu \right)e^{-\left(\beta_{n}^{2}+\nu \right)t}}{\left(\beta_{n}^{2}-\alpha +\nu \right)\left(\beta_{n}^{2}+\nu \right)}\right)\cos\frac{\beta_{n}}{\sqrt{\kappa }}x\right] \end{array}$$

where the thermal diffusivity, the diffusion rate coefficient, and $\beta_{n}$ are defined by Eqs. (7), (8), and (9), respectively:

(7) $$\kappa =\frac{k}{\rho c}$$

(8) $$\nu =\frac{hs}{\rho A\;c}$$

(9) $$\beta_{n}=\frac{n\pi }{L}\sqrt{\kappa }\;\;\;\;\;\;\;\text{for}\;n=1,2,3,\;.\;.\;.$$

#### 2.2.5 Parameter Uncertainty Calculation

The variable uncertainty is calculated for each parameter estimation performed. This is done by first calculating the mean standard error of the model to the data. The error was was assumed to be linear so that it could be estimated using the Jacobian matrix to form the covariance matrix. The individual errors could then be calculated by using the diagonal elements and the standard deviation [10]. A 95% confidence interval also used in calculating the error of the individual parameters.

## Ethics Statement

All the authors hereby declare that all the experiments were conducted while maintaining all ethical rules and regulations. None of the studies included humans or animals.

## CRediT authorship contribution statement

**Lauren B. Tomanek:** Conceptualization, Methodology, Software, Writing – original draft. **Daniel S. Stutts:** Conceptualization, Methodology, Software, Supervision, Writing – review & editing.

## Declaration of Competing Interest

The authors declare that they have no known competing financial interests or personal relationships which have, or could be perceived to have, influenced the work reported in this article.
